# Research Progress of Food-Derived Antihypertensive Peptides in Regulating the Key Factors of the Renin–Angiotensin System

**DOI:** 10.3390/nu17010097

**Published:** 2024-12-29

**Authors:** Xinyu Yao, Xinyi Cao, Liang Chen, Wang Liao

**Affiliations:** 1 Key Laboratory of Environmental Medicine and Engineering of Ministry of Education, Department of Nutrition and Food Hygiene, School of Public Health, Southeast University, Nanjing 210009, China; xinyu_yao@foxmail.com (X.Y.); 220214004@seu.edu.cn (X.C.); 2 Public Service Platform of South China Sea for R&D Marine Biomedicine Resources, The Marine Biomedical Research Institute, Guangdong Medical University, Zhanjiang 524023, China; cliang@gdmu.edu.cn

**Keywords:** antihypertensive peptides, the renin–angiotensin system, mechanisms of actions

## Abstract

Food protein-derived antihypertensive peptides have attracted substantial attention as a safer alternative for drugs. The regulation of the renin–angiotensin system (RAS) is an essential aspect underlying the mechanisms of antihypertensive peptides. Most of the identified antihypertensive peptides exhibit the angiotensin-converting enzyme (ACE) inhibitory effect. In addition, artificial intelligence has improved the efficiency of ACE inhibitory peptide identifications. Moreover, the inhibition of renin and blockade or down-regulation of angiotensin type I receptor (AT1R) have also been demonstrated to be effective intervention strategies. With the identification of the ACE2/Ang (1–7)/MasR axis, activation or up-regulation of angiotensin-converting enzyme 2 (ACE2) has also emerged as a new intervention pathway. This review summarizes the research progress of antihypertensive peptides in intervening with hypertension from the perspective of their properties, sources, and key factors. The objective of this review is to provide theoretical references for the development of antihypertensive peptides and the explorations of the molecular mechanisms.

## 1. Introduction

Hypertension is considered as one of the main risk factors for cardiovascular diseases. With global age-standardized prevalence rates remaining stable, the number of patients increased from 331 million women and 317 million men in 1990 to 626 million women and 652 million men in 2019 [1]. In addition, the hypertension guidelines issued by the American Heart Association lowered the hypertension diagnostic threshold from 140/90 mmHg to 130/80 mmHg, which means that the prevalence of hypertension will be significantly higher than previously reported [2]. Nowadays, thiazide diuretics (hydrochlorothiazide), angiotensin-converting enzyme inhibitors (captopril), angiotensin II receptor blockers (losartan potassium), beta-blockers (metoprolol), and calcium channel blockers (nifedipine) are commonly used pharmaceutical drugs for hypertension therapy. Despite the potent antihypertensive effect of these drugs, the duration of efficacy might be short and adverse reactions such as dry cough, edema, palpitation, headache, rapid pulse, dyspnea, dizziness, and renal function impairment [3,4] are always accompanied by the prolonged use of these drugs.

In recent years, there has been a growing emphasis on health-promoting ingredients from natural sources as an alternative to chemical antihypertensive drugs [5]. Food protein-derived bioactive peptides have attracted increasing attentions in functional foods due to their advantages of high bioactivity, low toxicity, and easy metabolism in the human body [6]. A survey of 9600 adults in Iran revealed that a more frequent intake of proteins, especially plant proteins, is more favorable for lowering blood pressure and exerting cardioprotective effects [7]. A meta-analysis on milk protein-derived bioactive peptides showed significant effect of lactotripeptides (VPP and LPP) in reducing both systolic and diastolic blood pressure (SBP and DBP) [8]. The dipeptide VY, from sardines, has been demonstrated to reduce blood pressure in mildly hypertensive or normotensive subjects, and no adverse effects were observed [9,10]. Despite much evidence of the effectiveness of various peptide sequences in clinical trials, their use in prophylaxis or therapy is not widespread. In the process of commercializing antihypertensive peptides, safety also requires more scrutiny.

In terms of blood pressure regulation mechanisms, the renin–angiotensin system (RAS) is the most important blood pressure regulation system in humans and other mammals; the regulation of key factors in the RAS has become a research direction of great interest in the treatment of hypertension [6]. Food-derived antihypertensive peptides exert their antihypertensive effects via regulating the main components of the RAS. In addition to the common antihypertensive mechanisms of inhibiting the activity of angiotensin-converting enzyme (ACE) and renin, blockade or down-regulation of angiotensin type I receptor (AT1R) has been a key strategy in the development of antihypertensive drugs [11]. Furthermore, due to the discovery of angiotensin-converting enzyme 2 (ACE2), activation of the ACE2/Ang (1–7)/MasR axis became a new target for food-derived antihypertensive peptides [12]. Classical methods of structure–activity relationship studies have several limitations in predicting and characterizing antihypertensive peptides. Researchers have begun to explore the use of artificial intelligence algorithms to construct and optimize structure–activity relationship models. Taking into account the above research background, we reviewed the research progress of food-derived protein antihypertensive peptides in intervening with hypertension from the perspectives of their properties, sources, and key factors for targeted regulation of the RAS, as well as the safety profile of antihypertensive peptides. Other signaling regulatory pathways with potential are also discussed. This review aims to provide a theoretical reference for the development of antihypertensive peptides and the exploration of their molecular mechanisms.

## 2. The RSA

In 1898, Tigerstedt et al. [12] extracted a heatable substance from rabbit kidneys and found that it had an antihypertensive effect, which was subsequently recognized as “renin”. Thereafter, the classical RAS was finally established with the discovery of angiotensin (Ang), angiotensin I (Ang I), ACE, and angiotensin II (Ang II) [13]. In the RAS, Ang secreted by the liver flows into the plasma and is hydrolyzed in the presence of renin secreted by the paraglomerular apparatus to Ang I. Ang I, a decapeptide with no pressor-raising ability, is catalyzed by ACE by cutting off the two amino acids at the C-terminal end and is converted to the octapeptide Ang II, which has a strong pressor-raising effect [14]. On the one hand, Ang II is able to bind to AT1R to cause vascular smooth muscle cell (VSMC) contraction and also stimulate aldosterone release to cause sodium and water retention in the kidneys, leading to an increase in blood pressure [15]. On the other hand, Ang II induces vasodilation, NO release, and inhibits cell growth by binding to angiotensin type II receptor (AT2R) [16].

**Figure 1:**
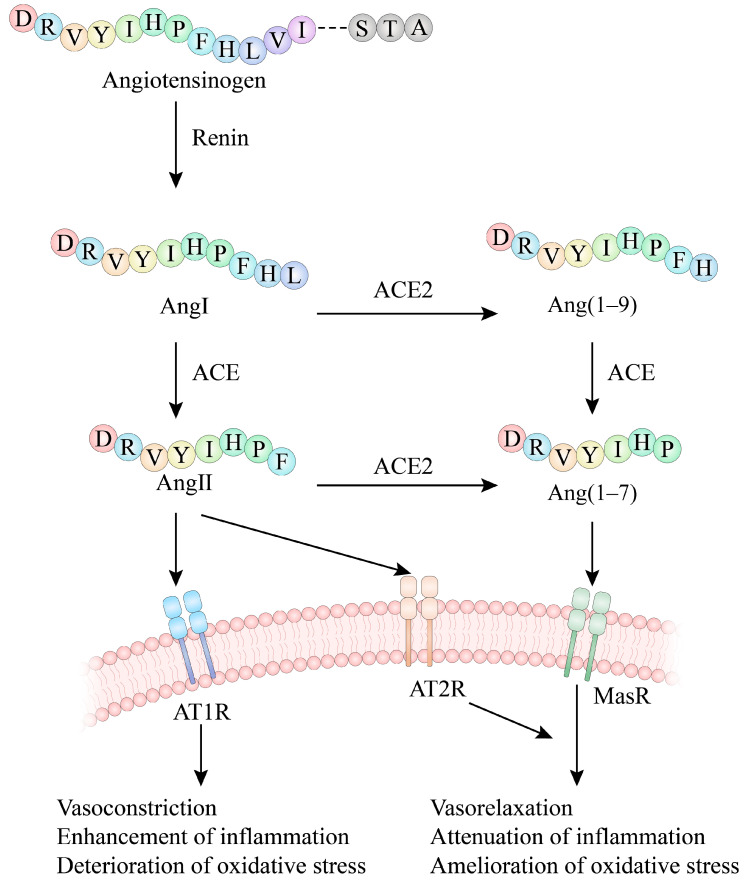
The role of ACE, renin, ACE2, and AT1R in the RAS.

With the identification of ACE’s homolog, ACE2, it was discovered that ACE2 cleaves the C-terminal phenylalanine of Ang II to form angiotensin 1–7 (Ang (1–7)) [17]. In contrast, Ang (1–7) binds to the G protein-coupled receptor (Mas receptor, MasR) and antagonizes Ang II-induced vasoconstriction [18]. In addition, ACE2 catalyzes the loss of a peptide from the C-terminus of Ang I to form the nine-peptide angiotensin 1–9 (Ang (1–9)) [16]. The generation of Ang (1–9) reduces the substrate of ACE, which generates Ang (1–7) in response to ACE [19]. Ultimately, the ACE2/Ang (1–7)/MasR axis was identified as a counter-regulatory pathway of the ACE/Ang II/AT1R axis, which is similarly crucial for regulating blood pressure. Thus, ACE2 activators can exert antihypertensive effects by increasing Ang (1–7) expression [20]. For example, xanthone (XNT) doubled ACE2 activity in the experiment. SHRs treated with XNT showed a significant reduction in mean arterial pressure (MAP), heart rate (HR), and increased cardiac function. Diminazene aceturate (DIZE) was reported as a potential ACE2 activator in a study using the tridimensional structure of ACE2 to dock a library containing FDA-approved drugs that showed protective effects in the heart, blood vessels, kidneys, and biliary tree. It also exerted beneficial effects on pulmonary hypertension and ischemia-induced cardiac dysfunction through activation of the ACE2/Ang (1–7)/MasR axis [21,22]. Mas receptor agonists also have potential for treating hypertension. A Mas receptor agonist called AVE0991 has shown antihypertensive effects in experiments. It stimulated the release of NO, exerted a vasodilatory effect, inhibited hypertension and organ damage, strengthened endothelial function, and exhibited a dose-dependent antihypertensive effect in certain types of hypertensive rats [20] (Figure 1).

## 3. Sources and Effects of Food-Derived Antihypertensive Peptides

Food-derived bioactive peptides are peptides formed from sequences of amino acids present in proteins that express biological activity [23]. They typically contain from 2 to 20 amino acid fragments from food and exhibit specific types of biological activity depending on the amino acid sequence, length, and configuration. They may have regulatory functions in human systems that go beyond the nutritional value of the parental protein. In addition to lowering blood pressure, antioxidant, antimicrobial, immunomodulatory, cholesterol-lowering, and antidiabetic effects have also been reported [24]. Food protein-derived antihypertensive peptides come from a wide range of sources, which has become a research hotspot in the field of nutrition and health.

Animal-derived antihypertensive peptides are mainly derived from dairy products, eggs, and muscles. Among them, IPP and VPP are the most successful antihypertensive peptides that have been exploited. They have antihypertensive effects in both spontaneous hypertension rats (SHRs) and humans and have been successfully used in commercial products such as *Calpis* (sour milk) and *Evolus* (fermented milk) [25,26]. Wongngam et al. [27] isolated the VSKRLNGDA peptide from alkaline protease-hydrolyzed chicken blood cells; SHRs fed the peptide orally (50 mg/kg body weight) showed reduced SBP by 41.83 mmHg 12 h later. After 4 weeks of feeding, blood pressure continued to drop. Egg white is rich in amino acids; egg white-derived peptides such as ITKPNDVYS, IRW, and TNGIIR have been shown to have antihypertensive effects in vivo for SHRs [11,28,29]. In addition, abundant marine products such as halibut [30], oysters [31] and tilapia [32] are also good sources of antihypertensive peptides.

Plant proteins such as grains, legumes, fruits and vegetables, and herbs and spices may also have a role in blood pressure reduction. Cocoa protein reduced SBP and DBP by 5% and 7%, respectively, (*p* < 0.001) in rats fed a high-fat diet [33]. Zou et al. [34] evaluated wheat bran protein hydrolysates for ACE and renin inhibition in vitro, then verified their ability to lower blood pressure in vivo. The soy-derived peptide FGSF had in vivo antihypertensive activity, with a maximum reduction in SBP of 21.95 mmHg after oral administration to SHRs [35].

**Figure 2:**
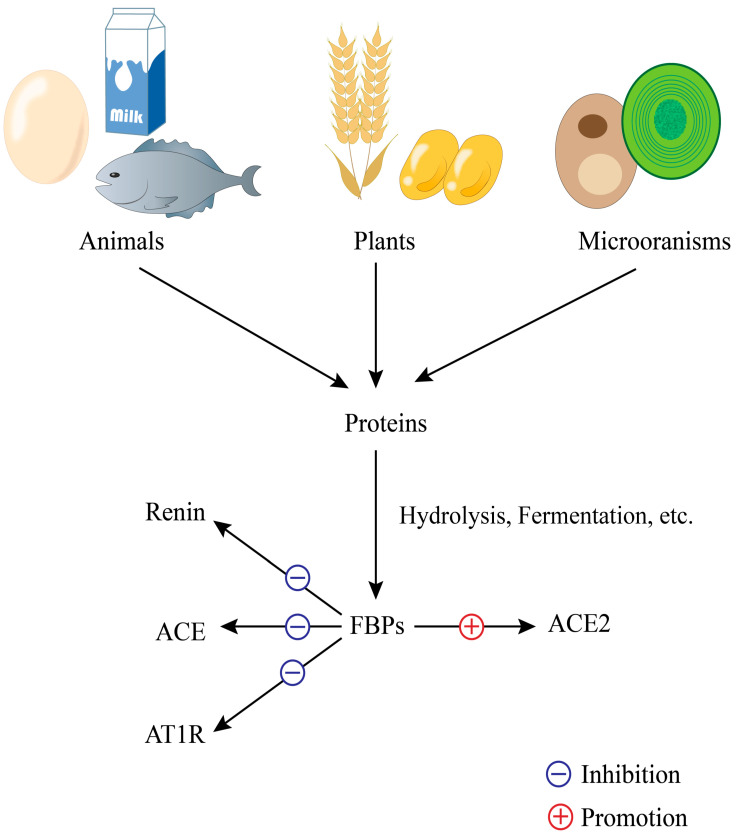
Sources of food-derived antihypertensive peptides and the involved pathways in regulating blood pressure.

Microorganisms are also a source of hypotensive peptides. The protein content of yeast extracts ranges from 50–70%; Mirzaei et al. [36] extracted the decapeptide YGKPVAVPAR and the nonapeptide LPESVHLDK, which were verified for their ability to inhibit ACE in vitro. Antihypertensive peptides extracted from spent brewer’s yeast had an IC50 (half maximal inhibitory concentration) of 84.2 μg/mL for fractions with molecular weights < 3 kda and maintained stable activity in the gastrointestinal system in vitro [37]. Zheng et al. [38] found that two peptides, IQP and VEP, from Spirulina could reduce SHRs’ blood pressure by down-regulating the ACE/AngII/AT1R axis and up-regulating the ACE2/Ang (1–7)/MasR axis (Figure 2).

## 4. Food-Derived Antihypertensive Peptides Regulating the Key Factors of RAS

### 4.1. ACE Inhibition

ACE is a zinc ion (Zn^2+^)-dependent dicarboxypeptidase located mainly in the cell membrane, and its catalytic site consists of three subunits, S1, S2 and S1’ [6]. The different types of ACE inhibition are categorized as competitive inhibition, noncompetitive inhibition, uncompetitive inhibition, and mixed competitive inhibition. The mechanism of competitive inhibition is that the ACE inhibitory peptide competes with Ang II for the same binding site of ACE, reducing the affinity between the enzyme and the substrate. Most of the known ACE inhibitory peptides follow this mechanism [39,40]. The noncompetitive inhibitory peptide binds to an essential group outside the ACE active center, inactivating the inhibitor–enzyme complex. In uncompetitive inhibition, the ACE inhibitory peptide binds to ACE only when the substrate binds to ACE [40]. Moreover, ACE can regulate blood pressure by acting on the kallikrein–kinin system (KKS). Bradykinin in the KKS acts on blood vessels, causing vasodilation and lowering blood pressure in the body. In the presence of ACE, bradykinin is hydrolyzed to inactive fragments, contributing to an increase in blood pressure. Thus, ACE inhibitory peptides reduce bradykinin inactivation and lead to lower blood pressure [41].

ACE inhibitory peptides are the most widely studied among food protein-derived antihypertensive peptides. Their activities are closely related to molecular weight, size, amino acid species, sequence structure and hydrophobic amino acid content, as well as C-terminal and N-terminal amino acids. Typically, small peptides with low molecular weights have higher hypotensive activity. Sonklin et al. [42] performed ultrafiltration membrane analysis of mung bean protein hydrolysate hydrolyzed by pineapple protease, and found that the IC50 values of the enzymatic products with molecular weights greater than 10 kda, 5–10 kda, 1–5 kda, and less than 1 kda were 0.68 ± 0.02 mg/mL, 0.58 ± 0.02 mg/mL, 0.64 ± 0.01 mg/mL, and 0.50 ± 0.01 mg/mL (*p* < 0.05). This indicates that the tendency of ACE inhibitory activity increases as the molecular weight of the enzymatic product decreases. Mirzapour et al. [43] also demonstrated that amygdalin hydrolysates with molecular weights less than 3 kda had higher ACE inhibitory activity (86.7%) compared to the higher molecular weight fractions. The C-terminal and N-terminal amino acids of ACE inhibitory peptides are another major factor affecting their activity [42]. Research has shown that peptides exhibit higher ACE inhibitory activity when the penultimate amino acid at the C-terminus is a valine, isoleucine, alanine, proline, tryptophan, or phenylalanine residue, or when the most terminal amino acid at the C-terminus is a tyrosine, tryptophan, phenylalanine, arginine, leucine, isoleucine, alanine, or methionine residue [44]. Of these, proline has the highest frequency of occurrence [45]. In addition, N-terminal aliphatic amino acids (glycine, alanine, isoleucine, leucine, and valine) can facilitate the interaction between the peptide and ACE, thereby enhancing the activity of ACE inhibitory peptides [46]. Of these, leucine has the highest frequency of occurrence [45]. The hydrophobic and hydrophilic properties of ACE inhibitory peptides are closely related to their activity. C-terminal hydrophobic amino acids can significantly increase ACE inhibitory activity [6,45]. Bougatef et al. [47] fractionated *Chelidonichthys lucerna* skin protein hydrolysates using high performance liquid chromatography (HPLC). In the two fractions with the most potent ACE inhibitory activity, peptides containing hydrophobic amino acids at the C-terminus accounted for 42.8% and 71% of the total number of peptides. A large percentage of hydrophobic amino acids, some as high as 100%, was observed in the different sequences of ACE inhibitory peptides listed by Huang et al. [48].

### 4.2. The Structure–Activity Study of ACE Inhibitory Peptides

**Figure 3:**
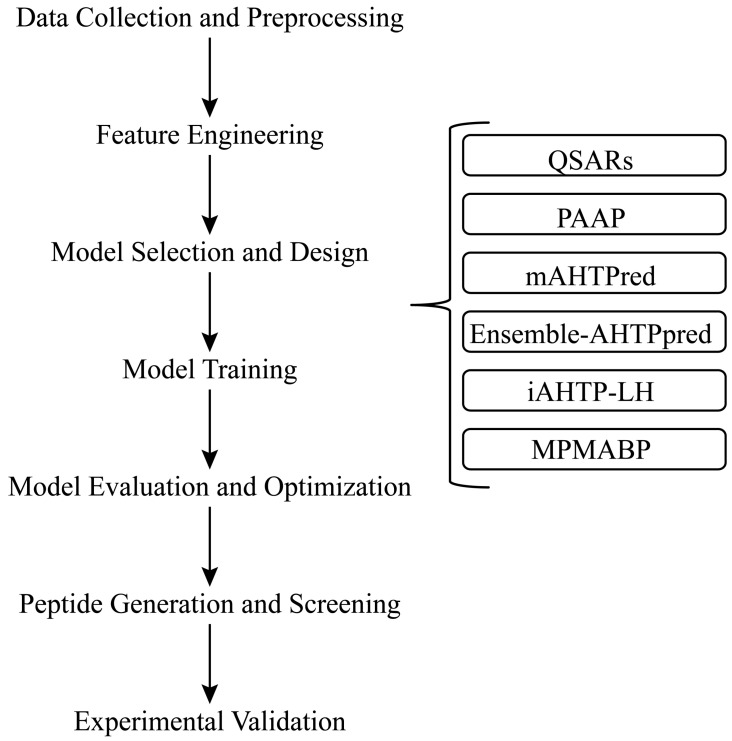
Identification of food-derived antihypertensive peptides based on artificial intelligence algorithms. The collection of relevant data and extraction of valid features from the database is the first step. Secondly, the model is selected and designed according to the requirements and the model is trained. The algorithm models that have been used include QSARs, PAAP, mAHTPred, Ensemble-AHTPpred, iAHTP-LH, MPMABP, etc. Then, the model is evaluated and optimized. Using the trained model, possible peptides are generated and screened. Finally, the activities of the peptides output from this process are experimentally tested to validate their antihypertensive peptide activity.

As compared with traditional structure–activity relationship research, the application of artificial intelligence algorithms to construct and optimize structure–activity relationship models improved the accuracy and efficiency of antihypertensive peptide predictions (Figure 3), which could facilitate the identifications of novel ACE inhibitory peptides and provide new options for the treatment of hypertension. Manavalan et al. [49] developed a new meta-predictor, mAHTPred, for the prediction and identification of antihypertensive peptides, which exhibited better prediction performance than existing methods on both benchmarking and independent datasets. However, mAHTPred has limitations in feature selection and algorithm choice. Despite using various feature encodings and machine learning algorithms, many potential features remain unexplored and redundant features may still affect model performance. Kalyan et al. [50] introduced a machine learning-empowered web server that predicts whether food-derived peptides have the ability to inhibit ACE. The web server can accept FASTA format or UniProt ID as input, perform digestion and screening, and output peptides with ACE inhibitory activity and their details. The regression decision tree model can accurately predict the IC50 values of peptides. The molecular docking results show the binding site and key residues of the peptide to ACE.

Li et al. [51] proposed a deep learning method (MPMABP) based on a convolution neural network (CNN) and bi-directional long short-term memory (Bi-LSTM) for recognizing multiple activities of bioactive peptides. MPMABP uses a CNN and Bi-LSTM to extract multi-scale and semantic features from peptide sequences, enabling it to capture complex patterns in the analyzed peptide sequences. The use of ResNet structures to preserve information and avoid information loss allows it to maintain good performance when dealing with long sequences. MPMABP also allows the study of the distribution of amino acids in different types of biologically active peptides; for example, proline tends to be found in antihypertensive peptides. However, its utility in predicting long-sequence peptides remains to be validated on larger datasets. Liao et al. [52] constructed a deep learning model using a long short-term memory (LSTM) algorithm. The model was validated with 54 peptides with in vitro ACE inhibitory IC50 values and in vivo antihypertensive effects. The results showed that the model had a high prediction accuracy. Additionally, 20 peptides were randomly synthesized and their IC50 values were determined experimentally using HPLC. Then they were compared with the predicted values from the model, which demonstrated that the model exhibited prediction efficiency. These AI algorithms build predictive models based on sequence information that utilize different feature representations and machine learning algorithms to extract and learn feature vectors of peptide sequences to classify or regress new peptide sequences.

While AI methods demonstrate clear advantages over traditional SAR approaches, they are not without limitations. Traditional SAR research often incorporates detailed experimental validation of peptide activity, providing insights into physicochemical properties and bioavailability. In contrast, AI-based methods heavily depend on the availability of high-quality datasets, which may lead to overfitting and poor performance on unseen peptides. The current AI models predominantly rely on in vitro data without considering the in vivo pharmacokinetics and pharmacodynamics of peptides. This affects the biological relevance of the predictions, particularly for therapeutic applications. Future improvements in AI-based models could address these problems by integrating in vivo activity data and employing hybrid approaches that combine traditional SAR knowledge with machine learning techniques, enhancing the biological relevance and practical utility of these predictive tools.

### 4.3. Renin Inhibition

Evidence suggests that prolonged use of antihypertensive drugs with ACE inhibition leads to Ang I accumulation and activation of other pathways such as chymotrypsin catalysis. Drugs resulting in the completion of Ang I to Ang II conversion independently of ACE would make antihypertensive drug therapy a failure. Direct inhibition of renin can prevent this situation for more effective lowering of blood pressure [53,54].

Renin is the rate-limiting enzyme at the start of the RAS chain reaction, consists of 350 amino acid residues, and is of high specificity. Human renin has two β-sheet domains, of which Asp38 and Asp226 are catalytically essential residues that form a catalytic duplex for hydrolyzing peptide bonds. The active site is formed by the junction of two structural domains with four subunits S1, S3, S1’, and S2’ [55]. For example, the FNLPILR peptide extracted from amaranth establishes two main hydrogen bonds, one to the acid group of renin Glu103 through the amino group of the side chain of the R residue and the other to the carboxyl group of renin Asp215 through the α-amino group of L. It is a competitive inhibitory peptide [56]. Fu et al. [57] analyzed the kinetics of renin inhibition by the Poria-derived peptides WG and PRY, confirming that the mechanism of inhibition is mixed. It has been observed that structural features with low molecular weight, hydrophobic amino acids at the N-terminal end, and bulky amino acids at the C-terminal end favor the inhibition of renin [53,57,58]. Ji et al. [59] found that of all the flaxseed peptides, the LW peptide with this profile had the strongest binding to renin. Baba et al. [60] molecularly docked peptides PVAAAPVM and LRPFL with renin, and the results of this study were consistent with the presence of aliphatic amino acids at the N-terminal end of renin-inhibiting peptides, in which the peptides PVAAAPVM and LRPFL were predicted to be potential renin-inhibiting peptides. Renin inhibitory activity is directly related to the hydrophobicity of the peptide. Olagunju et al. [61] separated pigeon pea protein hydrolysate into 18 fractions using reversed-phase high performance liquid chromatography (RP-HPLC), and the fractions with strong hydrophilicity had no measurable renin inhibitory activity, suggesting that the strong hydrophilicity of the peptides does not enhance their interaction with renin proteins. Conversely, hydrophobicity contributes to the renin-binding capacity of fractionated peptides. The SSYYPPFK peptide extracted from ruderalis by Zheng et al. [62] exhibited relatively high renin inhibitory activity (28.06–47.59%) with a high content of hydrophobic amino acid residues. The results of studies on the effect of molecular weight size on the inhibitory effect of renin vary, probably due to the fact that the inhibitory activity is more influenced by the sequence and structure of the peptide and by the protease used for hydrolysis [34,53,63,64].

Probably due to the fact that renin possesses a more folded protein conformation, renin inhibition is generally lower compared to ACE [42,65]. However, inhibition of renin alone may not prevent ACE from catalyzing bradykinin hydrolysis. Therefore, simultaneous inhibition of renin and ACE is an effective therapy for controlling hypertension [54]. LGF and GLFF peptides extracted from *moringa oleifera* proteins by Ma et al. [66] displayed good dual inhibitory activity against ACE and renin with IC50 values of LGF being 0.29 ± 0.13 mM and 1.88 ± 0.08 mM and GLFF being 0.31 ± 0.04 mM and 2.80 ± 0.08 mM for ACE and renin, respectively. In the in vivo model, LGF and GLFF significantly reduced SBP (19.4 mmHg, 18.2 mmHg) and DBP (12 mmHg, 13.8 mmHg) in SHRs. LGF and GLFF peptide transmembrane transport experiments and simulated gastrointestinal digestion experiments showed that they can resist gastrointestinal digestion in their complete form, which has the potential for the treatment of human hypertension. Rapeseed protein-derived LY, RALP, and GHS were identified as potent ACE and renin inhibitory peptides. After feeding SHRs with LY, RALP, and GHS for five weeks, the SBP of SHRs was reduced by 41 mmHg, 31 mmHg, and 28 mmHg, respectively, and the mRNA and protein expression of ACE and renin were significantly reduced [67]. The SNAGGGGGGGGP peptide derived from cocoa has the highest theoretical affinity for both ACE and renin (−9.1 kcal/mol and −3.9 kcal/mol). Oral administration of cocoa protein decreases blood pressure in rats consuming a high-density lipoprotein diet [33]. In addition, inhibition of renin and ACE has been demonstrated in hydrolysates of rice [68], wheat bran [34], and pigeon pea [65], which have the potential for the discovery of dual inhibitory peptides (Table 1).

### 4.4. AT1R Blockade and Down-Regulation

AT1R belongs to the G protein-coupled receptor superfamily that forms alpha helices in the lipid bilayer of cell membranes. Human AT1R has 359 amino acids and its main physiological functions are mediated by Gq-mediated phospholipase C activation, phospholipid hydrolysis, and Ca^2+^ signaling [73]. Blocking AT1R or downregulating AT1R protein expression likewise served to lower blood pressure without the side effects of ACE inhibition. The lactoferrin-derived peptide RPYL was one of the first peptides identified to inhibit the binding of Ang II to AT1R [74]. Since then, a variety of peptides capable of downregulating or blocking AT1R have been identified in foods.

Fan et al. [75] prepared chicken muscle hydrolysate, which when orally administered to SHRs for 20 days led to a significant reduction in blood pressure. Antihypertensive mechanism studies showed that the high-dose group was able to reduce circulating levels of Ang II (25.0 to 5.7 pg/mL) and down-regulate AT1R expression (35%). The VSKRLNGDA peptide from chicken blood cells was also able to inhibit AT1R expression [27]. Coronado-Cáceres et al. [33] found by bioinformatics and in vitro analysis that cocoa peptides strongly interacted with AT1R, of which 13 peptides had a higher affinity (−8.3 kcal/mol) compared to the drug Losartan, with the TLGNPAAAGPF peptide having the highest affinity value.

Cell migration is one of the pathogenic mechanisms leading to hypertension. Liao et al. [76] found that the anti-migratory activity of IRW in Ang II-stimulated A7r5 cells was dependent on AT1R, rather than MasR. TNGIIR peptides, also from egg white, were able to inhibit ACE and AT1R mRNA expression in SHRs and significantly reduced Ang II concentrations in serum [29]. Chen et al. [11] identified an AT1R down-regulating peptide, ITKPNDVYS, from egg white hydrolysate, which reduced the protein level of AT1R in A7r5 cells. It is hypothesized that ITKPNDVY may directly bind to and regulate the expression of AT1R, or downregulate AT1R by activating PPAR-γ signaling or inactivating the NF-κB pathway. It was further shown that egg whites have the potential to search for AT1R down-regulated peptides. However, due to the paucity of the literature, it is currently difficult to establish a relationship between structure and function.

### 4.5. ACE2 Activation and Up-Regulation

ACE2 is the homologous carboxypeptidase of ACE and a type I transmembrane glycoprotein containing 805 amino acids with an amino terminus that shares 42% sequence homology with the corresponding structural domain of ACE. However, due to differences in active centers, ACE2 activity is usually not fully antagonized by typical ACE inhibitors such as captopril [77]. ACE2 catalyzes the generation of the vasodilator Ang (1–7) from Ang II, thereby counteracting the effects of ACE [18]. Thus, activating ACE2 in the cardiovascular system provides another way to control hypertension.

IRW is a classical ACE2-activating peptide originally discovered as an ACE inhibitory peptide in egg-white ovotransferrin [78]. Oral IRW in SHRs can significantly lower blood pressure. However, the activity of plasma ACE was not affected, while Ang II levels were significantly reduced [28]. Subsequently, Majumder et al. [79] found by RNA-seq transcriptional analysis that IRW could reduce blood pressure by increasing mRNA levels of ACE2 in SHRs mesenteric arteries. Further studies showed that IRW is able of activating ACE2 in various systems. Up-regulation of ACE2 leads to a decrease in Ang II levels and an increase in Ang (1–7) formation, which exerts anti-inflammatory and vasodilatory effects via MasR, resulting in a decrease in blood pressure [80]. In vivo studies demonstrated that infusion of the MasR antagonist A779 decreased the antihypertensive effect of IRW on SHRs, providing further evidence of the ACE2/Ang (1–7)/MasR axis’s activation by IRW [81]. Recent studies have found that IRW can activate ACE2 via G protein-coupled receptor 30 (GPR30), clarifying the pathway of action for IRW [82]. Additionally, the bioavailability of IRW was found to be 11.7%, with half-lives of 7.9 ± 0.5 min for gavage and 28.5 ± 6.8 min for injection. And, the critical role of tryptophan and its metabolite, kynurenine, in IRW’s blood pressure-lowering effects has been identified [83].

The pea-derived peptide LRW, an isomer of IRW, exerts antioxidant, anti-inflammatory, and antiproliferative effects by up-regulating ACE2 in VSMC [84]. However, LRW failed to lower SHR blood pressure in vivo, possibly because of the lower transepithelial permeability and gastrointestinal stability of LRW [85]. Liao et al. [86] identified two ACE2 up-regulated peptides, LSDRFS and SDRFSY, from pea protein hydrolysate, which increased ACE2 expression in VSMC, but the in vivo roles need to be further investigated. Zhou et al. [87] found that soy protein hydrolysate significantly reduced Ang II-induced cell migration (129% to 92%), decreased ROS levels (2.22-fold to 1.45-fold), and increased NO levels (31.4 ± 0.7 μM to 43.7 ± 0.1 μM) in human umbilical vein endothelial cells. However, the ACE2 inhibitor MLN-4760 partially reversed these effects, indicating a potential regulatory role of soy protein hydrolysates on ACE2. Further studies showed that IVPQ and IAVPT peptides extracted from soy protein hydrolysates enhanced the activity of ACE2, and IVPQ up-regulated ACE2 protein expression in cells.

Chicken muscle protein is a good source of ACE2 up-regulating peptides. Fan et al. [88] found that spent hen protein hydrolysate (SPH-T) at a dose of 1000 mg/kg per day orally significantly lowered blood pressure in SHRs, as well as increased ACE2 expression in VSMC and enhanced antioxidant and anti-inflammatory activity, indicating its potential antihypertensive capacity in vivo. Four ACE2 up-regulated peptides, VKW, VHPKESF, VVHPKESF, and VAQWRTKYETDAIQRTEELEEAKKKK, were subsequently identified, resulting in a 0.52–0.84-fold increase in ACE2 expression [89]. Specifically, VVHPKESF increased cellular ACE2 activity through ACE2 upregulation rather than direct ACE2 activation. The antihypertensive effect of VVHPKESF may be due to the amelioration of inflammation, oxidative stress, and fibrosis caused by the upregulation of the ACE2/Ang (1–7)/MasR axis [75,90,91].

In fact, many peptides have both ACE2 up-regulation and ACE inhibition effects (e.g., LRW, IQP, VEP, RALP, GHS, LY, VKW) [38,67,84,89]. However, ACE2 activation is not a common feature of ACE inhibitory peptides. Not all ACE inhibitory peptides increase ACE2 protein levels in cells. Some of the identified ACE2 upregulating peptides (e.g., VHPKESF, VVHPKESF) also have no detectable ACE inhibitory activity in vitro [89,92]. This suggests that peptides have different structural requirements in the regulation of ACE and ACE2 (Table 2).

## 5. The Other Targets

Food protein-derived antihypertensive peptides can exert blood pressure-lowering effects using a variety of mechanisms. In addition to regulating key factors of the RAS, vasodilation was also induced by upregulating the expression of eNOS, COX, and prostaglandin receptor genes. Anti-inflammatory, anti-oxidative stress, improvement of endothelial function, vascular remodeling, blocking of L-type Ca^2+^ channels, modulation of the sympathetic nervous system, and inhibition of lipid accumulation may likewise play an antihypertensive role [18,94]. For example, rapeseed peptides have potential synergistic effects with captopril in vivo. Rapeseed peptides increased the blood pressure lowering capability of captopril by 9% and prolonged the duration of action by more than 20%. However, there was no reduction in ACE activity in organs with SHRs, but rather an increase in serum levels of NO (12.7%) and NOS3 (74.1%). Therefore, rapeseed peptides and captopril exert synergistic antihypertensive effects through pathways other than the RAS [95]. Chicken muscle protein-derived peptide VVHPKESF is able to reduce TNFα-induced inflammation and oxidative stress by inhibiting TNFR1 signaling in human vascular endothelial cells, with the potential for prevention and treatment of hypertension and cardiovascular diseases [96]. *Trichiurus lepturus* myosin hydrolysate (TMH) had a blood pressure-lowering effect in SHRs. After one month of administration in SHRs, Ang II levels decreased and bradykinin and NO levels increased. TMH also down-regulated the expression of ICAM-1 and VCAM-1, suggesting a strong anti-inflammatory effect. In addition, the expression of nitrotyrosine and collagen I was also decreased, suggesting a significant antioxidant effect of TMH, reflecting its multiple mechanisms of blood pressure lowering [97]. These discoveries provide new ideas for the study of food protein-derived antihypertensive peptides.

## 6. Potential and Challenges of Food-Derived Antihypertensive Peptides in Translations

Studies of food-derived antihypertensive peptides in humans have revealed their remarkable potential in the management of hypertension. Liao et al. [98] reviewed clinical trials on antihypertensive peptides derived from food proteins conducted between 2010 and 2021 and performed a meta-analysis. The study found that these peptides significantly reduced SBP (−3.28 mmHg) and DBP (−1.82 mmHg), with more pronounced effects observed in participants with a higher basal blood pressure. In addition, Asian participants, groups with a higher proportion of female participants, and participants with an average age over 50 responded more strongly to the peptides. A recent study compared the effects of hemp seed protein (HSP) and its hydrolysate-derived bioactive peptide (HSP+) with casein protein on hypertensive adults. HSP and HSP+ significantly reduced 24-h SBP and DBP, and the HSP+ showed superior efficacy. Additionally, these proteins were found to lower both ACE and renin activities and raise NO concentration in plasma compared with casein [99].

Nevertheless, Sato [100] pointed out that the concentration of food-derived peptides in human plasma tend to be much lower than the effective concentrations in in vitro assays, suggesting that their antihypertensive effects may rely on metabolites or indirect mechanisms. These findings further emphasize the importance of studying the responses of different populations to food-derived antihypertensive peptides and optimizing their bioavailability. Future research should build upon this understanding to explore the long-term effects of these peptides in various populations and develop tailored intervention strategies considering gender, age, and regional characteristics. This will facilitate the broader application of functional foods. Future research should focus on exploring the long-term effects of antihypertensive peptides in diverse populations and developing tailored intervention strategies considering factors such as gender, age, and regional characteristics, to facilitate the broader application of functional foods. Additionally, clinical trials should include larger cohorts with varied hypertension types and specific groups like pregnant women, the elderly, and individuals with comorbidities, to better evaluate their efficacy and safety.

## 7. Safety Assessment of Antihypertensive Peptides

In the commercialization of food-derived antihypertensive peptides, it is important to consider their potential health-promoting benefits but also to be wary of possible unsafe factors. Assessment of the toxicity of antihypertensive peptides is the basis for determining their safety. The most widely used milk-derived antihypertensive peptides have not been found to be toxic in toxicological experiments to date [101,102,103,104,105,106]. Safety assessments have mostly focused on Ile-Pro-Pro and Val-Pro-Pro. Several studies using randomized, double-blind, placebo-controlled designs have included healthy individuals, prehypertensive, and hypertensive patients. The results showed no adverse effects such as dry cough, rash, or dizziness, nor any abnormalities in the respiratory, gastrointestinal, neurological, or skin systems. Furthermore, these peptides have been shown to significantly reduce blood pressure, total cholesterol, and low-density lipoprotein cholesterol levels, while improving insulin sensitivity and the neutrophil-to-lymphocyte ratio [105]. Some plant-derived antihypertensive peptides, such as SAPPP, PLLK, and PPMWPFV, were predicted for potential security in silico, which showed them to be non-toxic. However, further in vivo experiments are required to ensure their safety and efficacy [107,108].

Clinical trials play a critical role in evaluating the safety and efficacy of antihypertensive peptides. These trials are conducted in phases: Phase 0 ensures safety using low doses in a small group of participants; Phase I focuses on safety in 20–100 volunteers over several months; Phase II assesses efficacy over several months to two years with randomized and blinded designs; Phase III evaluates effectiveness in thousands of participants over several years; and Phase IV, a post-marketing phase, examines long-term effects, cost-effectiveness, and potential quality-of-life impacts [105].

It is important to note that some peptides possess structural features similar to those of toxic peptides and exhibit biological activity. For example, Leu-Lys-Leu derived from sardines has shown inhibitory effects on ACE [109]. Certain bioactive peptides may trigger immune responses, leading to allergies or other adverse health effects [104,110]. This typically involves mediation by immunoglobulin E (IgE). Currently, peptides with ACE inhibitory activity have been shown to cause allergic reactions in humans. According to predictions from the BIOPEP-UWM database, the peptide sequence Phe-Val-Ala-Pro-Phe-Pro-Glu-Val-Phe-Gly-Lys extracted from casein hydrolysates has been identified as an allergenic peptide, which also displayed inhibitory effects on ACE but lacked in vivo data [102]. Protease hydrolysis, heating, irradiation, and high hydrostatic pressure are methods to disrupt the binding of IgE to epitopes, which can reduce the allergenicity of food allergens [111]. Furthermore, although short-term studies support the safety of food-derived antihypertensive peptides, data on the safety of long-term consumption of these peptides is still relatively limited. Another key consideration is their interaction with traditional medications. These interactions may either enhance or weaken the effects of the medications, leading to unstable blood pressure control.

## 8. Conclusions

The global rise of hypertension demands innovative solutions. Food-derived antihypertensive peptides hold immense potential for blood pressure regulation and cardiovascular health promotion. These peptides, sourced from plants, animals, and microorganisms, demonstrate diverse interactions with the RAS, including ACE inhibition, renin inhibition, ACE2 activation, and the AT1R blockade, among other pathways. Their clinical relevance lies in providing safer and more natural alternatives for antihypertensive therapy.

To translate these findings into clinical practice, the following aspects should be prioritized:

1. Although ACE2 up-regulatory peptides appear to share some of the same structural features with ACE inhibitory peptides, such as N-terminal hydrophobic and C-terminal aromatic or cyclic residues, as seen in IRW, LRW, IQP, VEP, LY, VKW, VHPKESF, and VVHPKESF [38,81,89], research on ACE2 up-regulated peptides remains limited. Future studies should explore structure–activity relationships to predict and construct potent ACE2-activating peptides. Such research can contribute to the development of novel therapeutic agents or functional foods targeting hypertensive patients, particularly those resistant to current ACE inhibitors.
2. Most current research focuses on in vitro antihypertensive activity with insufficient clinical validation. To facilitate clinical translation, future studies must evaluate the oral bioavailability, pharmacokinetic properties, and long-term safety of antihypertensive peptides. These efforts will enable the development of functional foods or nutraceuticals that can be seamlessly integrated into hypertensive patients’ treatment regimens, offering accessible, complementary solutions to conventional therapies.
3. Clinical trials on antihypertensive peptides often have small sample sizes, limiting their generalizability. Future studies should include larger cohorts with diverse hypertension types, genders, and specific populations, such as pregnant women, the elderly, and those with comorbidities, to better assess their efficacy and safety.
4. The prediction model of antihypertensive peptide activity based on artificial intelligence algorithms has great potential. However, it is necessary to consider not only the performance of antihypertensive peptides in the in vitro environment, but also the biological relevance to improve the confidence of the model.

## Figures and Tables

**Table 1:** Peptides with renin inhibitory effects.

Source	Peptide Sequence	Production (Enzymes)	Inhibitory Activity	Decrease in BP After Administration	References
Pigeon pea peptides	AGVTVS	Hydrolysis with pepsin and pancreatin	82%	-	[61]
Mung bean proteins	YADLVE	Hydrolysis with bromelain	97.06%	SBP decreased by 27 mmHg	[42]
Proteins in squid processing by-products	-	Hydrolysis with neutral protease	69.72 ± 1.16% (Simulated gastrointestinal digestion was reduced to 43.17%), IC50 = 1.47 ± 0.06 mg/mL	-	[69]
*Moringa oleifera* protein	LGF	Hydrolysis with alcalase	IC50 = 1.88 ± 0.08 mM	SBP decreased by 19.4 mmHg, DBP decreased by 12 mmHg	[66]
	GLFF		IC50 = 2.80 ± 0.08 mM	SBP decreased by 18.2 mmHg, DBP decreased by 13.8 mmHg	
Sesame seed protein	-	Hydrolysis with pepsin and pancreatin	75–85%	-	[70]
Amaranth proteins	FNLPILR	Hydrolysis with alcalase	IC50 = 0.41 mM	-	[56]
Naked oat globulin	SSYYPPFK	Hydrolysis with alcalase, flavourzyme, pepsin, and trypsin in sequence	28.06–47.59%	Effectively decreased SBP and DBP	[62]
Hempseed protein	WYT	Hydrolysis with different proteases (alcalase, flavourzyme, thermolysin, pepsin, and trypsin)	IC50 = 0.054 mM	-	[54]
Wheat bran protein isolate	<1 kDa fraction (NL, QL, FL, HAL, AAVL, AKTVF, TPLTR)	Hydrolysis with alcalase	75.19% ± 1.75%	SBP decreased by 35 mmHg	[34]
Rice protein isolate	-	Hydrolysis with pepsin	IC50 = 2.7 mg/mL	-	[68]
Rapeseed protein isolate	LY	Hydrolysis with alcalase	-	SBP decreased by 41 mmHg	[67]
	GHS			SBP decreased by 28 mmHg	
	RALP		IC50 = 0.97 mM	SBP decreased by 31 mmHg	[54,67]
Peanut protein	-	Hydrolysis with alcalase	IC50 = 1.78 mg/mL	-	[71]
Tartary buckwheat albumin	LFFR	Hydrolysis with pepsin	IC50 = 5.00 mM	-	[72]
	LGLLPYFR		IC50 = 10.19 mM		

“-” indicates that references did not provide the corresponding information for that column.

**Table 2:** Peptides with activating or up-regulating effects on ACE2.

Source	Peptide Sequence	Production (Enzymes)	ACE2-Related Activities	Decrease in BP After Administration	References
Rapeseed protein	RIY	Hydrolysis with subtilisin	Activated circulating/ aortic ACE2	Effectively decreased MBP and DBP	[92]
Chicken muscle protein	IKW	Hydrolysis with thermolysin			
Chicken muscle protein	VKW, VHPKESF, VVHPKESF, VAQWRTKYETDAIQRTEELEEAKKK	Hydrolysis with thermolysin	Increased ACE2 expression by 0.52–0.84-fold	-	[89,93]
Pea protein	LRW	Solid-phase synthesis	Increase ACE2 levels	Inability to lower blood pressure in SHRs due to its low gastrointestinal stability and trans-epithelial permeability	[84,85]
	LSDRFS, SDRFSY	Hydrolysis with a combination of thermoase and pepsin	A significant effect on up- regulating ACE2 expression	-	[86]
Soybean protein	IVPQ, IAVPT	Through peptidomics analysis and bioinformatics tools	IVPQ and IAVPT enhanced ACE2 activity and only IVPQ increased ACE2 protein expression in HUVECs	-	[87]
Ovotransferrin	IRW	Hydrolysis with proteases or peptidases	ACE2 activation depends on the G protein- coupled receptor 30 signaling cascade	Effectively decreased SBP and DBP, MBP decreased by 20.97 mmHg	[28,79,80,81]
Spirulina platensis	IQP, VEP	Hydrolysis with papain	Up-regulated the mRNA and protein levels of ACE2	Effectively decreased BP	[38]
Rapeseed protein isolate	RALP, GHS, LY	Hydrolysis with alcalase	Up-regulated the mRNA and protein levels of ACE2	Effectively decreased SBP	[67]

“-” indicates that references did not provide the corresponding information for that column.

## Abbreviations

RAS, renin–angiotensin system; ACE, angiotensin-converting enzyme; AT1R, angiotensin type I receptor; ACE2, angiotensin-converting enzyme 2; SBP, systolic blood pressure; DBP, diastolic blood pressure; Ang, angiotensin; Ang I, angiotensin I; Ang II, angiotensin II; VSMC, vascular smooth muscle cell; AT2R, angiotensin type II receptor; Ang (1–7), angiotensin 1–7; MasR, Mas receptor; Ang (1–9), angiotensin 1–9; XNT, xanthone; MAP, mean arterial pressure; HR, heart rate; DIZE, diminazene aceturate; SHRs, spontaneous hypertension rats; HSP, hemp seed protein; HSP+, hydrolysate-derived bioactive peptide; FBPs, food-derived bioactive peptides; KKS, kallikrein-kinin system; HPLC, high performance liquid chromatography; CNN, convolution neural network; SAR, structure–activity relationship; Bi-LSTM, bi-directional long short-term memory; LSTM, long short-term memory; RP-HPLC, reversed-phase high performance liquid chromatography; GPR30, G protein-coupled receptor 30; TMH, *Trichiurus lepturus* myosin hydrolysate; IgE, immunoglobulin E.
